# Multi-Functional Hybrid Terpolymer Thermosets Based on Thiols Bio-Based Epoxy and Benzoxazine Monomers

**DOI:** 10.3390/polym17172389

**Published:** 2025-09-01

**Authors:** Madalina Ioana Necolau, Elena Iuliana Biru, Elena Olaret, Horia Iovu

**Affiliations:** 1 Advanced Polymer Materials Group, National University of Science and Technology POLITEHNICA Bucharest, 060042 Bucharest, Romania; madalina.necolau@upb.ro (M.I.N.);; 2 Academy of Romanian Scientists, Ilfov 3, 050044 Bucharest, Romania

**Keywords:** bio-based thermosets, benzoxazine, epoxy, terpolymer, thiol-ene thermoset

## Abstract

Hybrid thermosetting terpolymers based on epoxidized linseed oil (ELO), eugenol-based benzoxazine monomer (EPB), and thiols (2SH and 3SH) were synthesized and studied by focusing on the effects of the thiol-bearing functionality over the final performances. The curing dynamics were monitored by differential scanning calorimetry (DSC) and Fourier transform infrared spectrometry (FTIR). FTIR results showed that the curing process takes place in multiple steps and depends on the concentration of thiol used as a crosslinker. At the same time, the complexity of the reactions that take place within each system was highlighted by the curing profiles from DSC. Dynamic mechanical analysis (DMA) and nanoindentation data revealed that the mechanical features of the terpolymers can be modulated to achieve high stiffness, as in the case where 2SH and 3SH thiols were used in 0.25 wt.% or increased flexibility where 1% thiol concentrations were employed. Higher crosslinking density for hybrid terpolymers in comparison with the epoxy/benzoxazine sample indicated a good compatibility between the monomers and the crosslinking agents and the formation of additional chemical bonds within the networks. The ternary samples demonstrated good thermal stability (up to 300 °C) and high residual mass (>25%), which make them suitable candidates as flame-resistant coatings.

## 1. Introduction

Thermosets represent one of the most valuable classes of polymeric materials with multiple industrial applications that play an important role in modern engineering and manufacturing. When polymerized, they form highly crosslinked networks with structural integrity and durability, making them valuable for a wide range of applications, such as automotive, aerospace, coatings, electronics, and construction [1,2,3,4,5,6,7].

Over the last decade, the development of novel thermosets with improved thermal resistance, mechanical properties, sustainability, and processability has become the focus of researchers [8]. Finding bio-based alternatives with balanced performances has the potential to diminish the carbon footprint of petroleum-based resins while addressing the environmental concerns brought by the depletion of these resources. Thus, in the actual context of increasing bio-based content of polymeric thermosets, different resources, such as natural phenols (eugenol, sesamol, vanillin, and guaiacol) [9,10,11,12,13,14,15], amines (furfurylamine and tyramine) [16,17,18], and oils (soybean, linseed, and corn) [19,20,21], have been successfully used to synthesize a wide variety of reactive monomers.

Polybenzoxazine resins stand as a representative material for this polymer class, as they exhibit superior properties and may be easily obtained by thermal-induced polymerization of benzoxazine monomers without the aid of any catalyst. These versatile monomers benefit from a tremendous design flexibility as a consequence of their facile synthesis through Mannich condensation between phenols, primary amines, and formaldehyde [22,23,24]. By using compounds with different functionalities and structures, the final performances can be easily tailored to meet specific requirements, such as high mechanical resistance, toughness, electrical insulating features, chemical resistance, or anticorrosive effects. However, there are still some limitations associated with these materials. Most benzoxazine monomers are solid by nature and need to be melted before processing. Their polymerization takes place at higher temperatures between 220 and 260 °C and they become brittle after curing [24,25]. Viscous benzoxazine monomers have recently been synthesized by using branched polyethyleneimine (PEI) alongside eugenol, sesamol, guaiacol, and vanillin [13,26,27,28]. Apart from their facile processability, the numerous free amino groups represent versatile reactive sites that can further be used to develop novel and advanced materials.

A possible strategy to overcome benzoxazine disadvantages would be blending with another thermoset. Copolymerization or development of multicomponent networks represents a valuable strategy through which properties can be modulated toward facile development of advanced materials. Up to now, benzoxazine has been used alongside urethane [29], epoxy [30,31], polydimethylsiloxane [32,33], thiols [34,35], benzimidazole [36], and ε-caprolactone [37]. Among these examples, epoxy represents the most suitable candidate to obtain blends due to its increased compatibility with benzoxazine. Epoxy resins exhibit some particular features, such as strong adhesion toward a variety of substrates, ease of processing and application, superior toughness, and well-regulated industrial applicability. Another important difference comes from the polymerization mechanism, which in this case requires a hardening agent, such as amine, anhydride, or carboxylic acid [38,39,40].

For instance, copolymerization of benzoxazine with different types of epoxy monomers will lead to significant modifications in both thermal and mechanical properties of the resultant hybrid networks in comparison with benzoxazine homopolymer. Bisphenol-A benzoxazine monomer and aromatic epoxy (EPON 826) led to a copolymer with shape memory activity and superior mechanical properties [41]. Best results were recorded for the sample where stoichiometric ratios of benzoxazine, epoxy, and crosslinking agent were used, the final network having a Tg of 120 °C. A similar shape memory effect was obtained in different studies, where benzoxazine was copolymerized with epoxy monomers based on epoxidized castor oil [42,43].

Patil et al. [44] synthesized a bio-based hybrid network by mixing eugenol-based benzoxazine monomers with conventional DGEBA and gallic acid epoxy monomers. The benzoxazine structure contained a free amino group, which acted as a crosslinker for the epoxy monomers, and the final performances were evaluated as a function of epoxy resin type. The bio-based system demonstrated higher Tg and superior thermal resistance with enhanced flame-resistant properties.

Thiols represent a valuable class of chemical compounds with an important role in thermoset chemistry. Their high reactivity toward numerous functionalities makes them suitable as crosslinking agents for systems containing epoxy, benzoxazine, alkenes, and even urethanes. Up to now, the use of thiols has been studied alongside epoxy resin and benzoxazines, and numerous advantages, such as decreasing the curing temperature and improving mechanical and thermal stability, were observed [45,46,47,48].

The present study aims to develop complex bio-based thermoset networks by combining the advantages of benzoxazine with epoxy monomers. The free amino groups present on the structure of eugenol-based benzoxazine monomers have the potential to chemically interact with the oxirane ring of epoxidized linseed oil, leading to a homogeneous network with high crosslinking density. At the same time, the potential of thiols as common crosslinking agents was assessed by studying their influence over polymerization temperature and network properties. It was already demonstrated that these functional groups exhibit a high reactivity toward both oxazine and epoxy moiety. Thiol groups facilitate ring-opening reactions under mild conditions and have the potential to improve toughness and flexibility of thermosets while acting as chain extenders at the same time. Thus, by combining benzoxazine with epoxidized oil chains and thiols, brittleness can be diminished, and the reactivity of the system can be improved, leading to novel hybrid copolymeric networks with unique features.

## 2. Materials and Methods

Eugenol-PEI Bz monomer (EPB) [27] and epoxidized linseed oil (ELO) [20] were previously synthesized and characterized by our team. Here, 2,2′-ethylenedioxy diethanethiol 95% (2SH), trimethylolpropane tris(3-mercaptopropionate) ≥ 95.0% (3SH), and ethylene glycol were used for analysis (EMSURE^®^ Reag. Ph Eur, Reag, Darmstadt, Germany). USP from Merck was employed for the contact angle measurements.

### 2.1. Synthesis Process of Hybrid Bio-Based Networks

**Figure 1:**
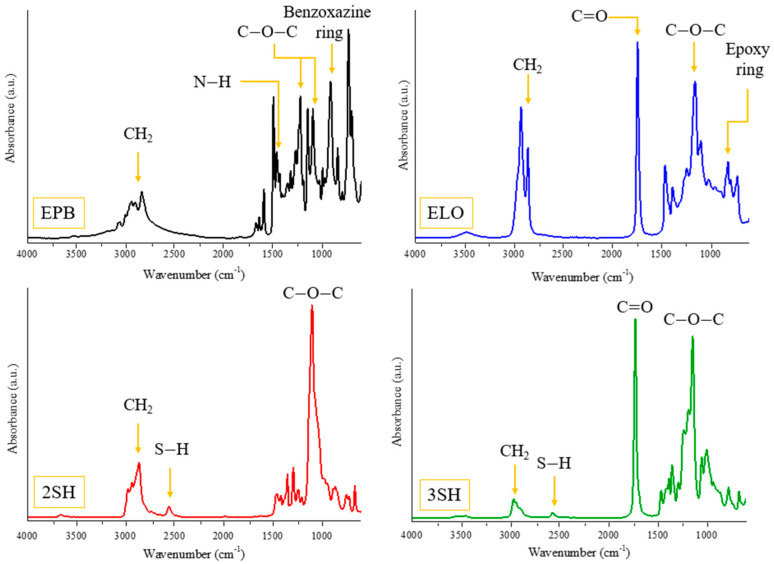
The FTIR spectra of the reactants used in the synthesis of hybrid networks.

In order to determine the effect of thiols in composition with benzoxazine and epoxy over the curing dynamics and network properties, the following formulations were synthesized, as described in Table 1. The chemical structures of the compounds used in the synthesis of the tricomponent systems are represented in Figure 1.

The hybrid networks were synthesized in two steps. Firstly, the eugenol-benzoxazine and epoxy monomers (1:1 wt.%) were mixed under a magnetic stirrer for 15 min at 60 °C to decrease the viscosity of the components and to facilitate homogenization. In the second step, the mixture was cooled down to room temperature before thiol incorporation. The composition of each formulation along with the abbreviations used within the manuscript are presented in Table 1.

After the thiol was added and homogenized, the mixture was poured in a Teflon mold and cured for 4 h at 180 °C, 1 h at 200 °C, and 1 h at 220 °C to ensure complete polymerization of both epoxy and oxazine functionalities. The curing program was established according to DSC results by considering the onset temperature of the exothermal transition.

### 2.2. Methods

Fourier transform infrared (FTIR) spectra were recorded on a Bruker Vertex 70 equipment using a platinum ATR module in 400–4000 cm^−1^ range with 4 cm^−1^ resolution and 32 scans. The reaction progress over time was investigated by focusing on the benzoxazine and epoxy signals observed at 915 and 840 cm^−1^. The FTIR spectra of the final materials were normalized by taking as reference a constant band (C=O ester group from 1743 cm^−1^). The reaction extent was computed by considering the intensity of epoxy and benzoxazine signals at a given reaction time in comparison with the intensity at the initial time (t = 0).

Differential scanning calorimetry (DSC) analysis was performed on a Netzsch DSC 204 F1 Phoenix equipment in the 20–300 °C temperature range under nitrogen atmosphere (20 mL/min flow rate). The measurements were conducted at heating rates of 5, 10, 15, and 20 °C/min and the apparent activation energy (Ea) of the curing processes was determined with the aid of Kissinger (Equation (1)) and Ozawa (Equation (2)) equations:

(1) $$\ln(\frac{\beta }{T_{p}^{2}})=\ln\frac{AR}{Ea}\;-\;\frac{Ea}{{RT}_{p}}\;$$

(2) $$\ln\beta =-1.052EaRT_{p}+C$$

where β is the heating rate (°C/min), T_p_ is the maximum temperature of the polymerization peak (K), A is the pre-exponential factor, R is the gas constant (R = 8.314 J/mol·K), Ea is the activation energy (kJ/mol), and C is a constant.

Thermogravimetric analysis (TGA) was performed using a Netzsch TG 209 F1 Libra equipment. Samples weighing ~10 mg were heated from 25 to 700 °C with a heating rate of 10 °C/min under nitrogen atmosphere. The limiting oxygen index (LOI) was determined based on residual mass after TGA by employing the Van Krevlen equation:

(3) $$LOI\left(\%\right)=17.5+0.4\times Residual\;mass(\%).$$

Dynamic mechanical analysis (DMA) was conducted on a TRITEC 2000 B equipment in single cantilever bending mode at 1 Hz frequency. Samples were analyzed in the temperature range of −60 to 100 °C with a heating rate of 5 °C/min. Based on the DMA results, the crosslinking density (CD) and the molecular weight between crosslinking points (Mc) were calculated for each hybrid network by employing the following equations:

(4) $$CD=\frac{E^{\prime }}{3RT},$$

where E′ represent the storage modulus in the rubbery region at T = Tg + 30, T corresponds with the absolute temperature in K, and R is the ideal gas constant (8.314 J/mol·K), and

(5) $$M_{c}=\frac{\rho RT}{CD}$$

where ρ is the polymer density, υe is the crosslinking density, R is the gas constant (R = 8.314 J/mol·K), and T is the absolute temperature in the rubbery region in K.

To evaluate the gel content of the crosslinked hybrid networks, dry samples (~500 mg, W_0_) were extracted in toluene (5 mL) for 24 h. After completion of the extraction process, the samples were dried in a vacuum oven at 60 °C for 24 h before weighing (W_1_). The gel content of the samples was then calculated using the following equation:

(6) $$\mathrm{Gel}\mathrm{content}\;(\%)\;=\;\frac{W_{1}}{W_{0}}\;\times \;100\%$$

Contact angle (CA) measurements were performed on a drop shape analyzer DSA100 from Krüss Scientific GmbH, Hamburg, Germany by employing a sessile drop method at room temperature. Water and ethylene glycol were used as the polar and nonpolar probing liquids, and the contact angle values were determined using the Young–Laplace equation in ADVANCE 1.7.2.1 software by registering 10 frames/s. The final results represent the average values for each sample. The determination of surface-free energies was calculated in the same software using the Young–Dupré and Fowkes equations, which considered the polar work of adhesion and the corresponding surface tensions of the liquids used for analysis.

Hydrolytic stability and degradation of the synthesized hybrid thermosets were evaluated by immersion in water, NaOH 1 M, and H_2_SO_4_ 1 M for 24 h at 60 °C. For each system, 3 specimens were tested and the adsorption degree (AD%) was calculated with the aid of the following formula:

(7) $$AD\;\left(\%\right)=(\frac{m_{f}-m_{0}}{m_{0}})\times 100\%$$

where m_0_ represents the initial mass of the specimen, while mf represents the final mass after the absorption.

For nanoindentation, mechanical properties at the microscale were obtained using a dynamic instrumented indentation system (G200 Nano Indenter system, KLA Instruments, Milpitas, CA, USA). The “G-Series DCM CSM Flat Punch Complex Modulus” method, implemented within NanoSuite 6.52.0 software, was used to determine the storage (G′) and loss (G″) moduli at a 10 µm indentation depth and 50 nm oscillation amplitude at a test frequency of 10 Hz. Seven tests were performed on each sample at room temperature using a diamond flat-ended cylindrical punch indenter with a punch diameter of 97.7 µm. Results were computed using Poisson’s ratio of 0.4 and were expressed as mean ± standard deviation.

In the statistical analysis, all experiments were performed in triplicate (n = 3) unless otherwise stated and the results were expressed as a mean ± standard deviation (SD). Statistical relevance was assessed using GraphPad Prism Software 8.0 (GraphPad Software Inc., San Diego, CA, USA), the one-way ANOVA method, and the Bonferroni post-test considering significant statistical differences for *p* < 0.05.

## 3. Results and Discussion

### 3.1. Structural Characterization of the Monomers

FTIR analysis was used to characterize the components of the system prior to hybrid network formation. The chemical structures of the components are provided in Supplementary Figure S1. Figure 1 presents the spectrum of the monomers employed in the synthesis, highlighting the most significant functional groups.

EPB oligomers display characteristic signals for the benzoxazine ring, such as out-of-plane bending vibrations at 915 cm^−1^, C–N–C symmetric stretching at 1146 cm^−1^, and asymmetric and symmetric stretching vibrations of C–O–C at 1220 and 1094 cm^−1^, respectively [27,49]. The peak from 1640 cm^−1^ corresponds with the C=C stretching of the allyl group from eugenol, while the signal from 1592 cm^−1^ is representative of skeletal vibrations of the aromatic rings [50]. Apart from that, characteristic signals for the amine functionalities present on the polyethyleneimine backbone are observed at 1272 and 1349 cm^−1^ and they correspond to C-N stretching vibrations [51]. The small peaks from 794 cm^−1^ correspond with out-of-plane wagging vibrations associated with N–H from primary amines, while the signal from 1464 cm^−1^ is associated with N–H bending [52,53].

The spectra for epoxidized linseed oil (ELO) show characteristic signals for the carbonyl groups (C=O stretching vibrations) from the esters included in the triglyceride backbone at 1743 cm^−1^. The peaks from 1462 and 1386 cm^−1^ correspond to the bending vibrations of methylene and methyl groups, while the signals from 1242, 1157, and 1097 cm^−1^ are associated with the asymmetric stretching vibrations of C–O–C groups. Additionally, the C–O–C bending signal related to the oxirane ring can be observed on the spectra at 840 cm^−1^ [54,55].

Analyzing the spectra of the 2SH and 3SH thiols, the presence of specific functional groups, such as S–H stretching and C–S stretching vibrations, can be observed at ~2560 and ~670 cm^−1^. The cluster peak around 2838–2971 cm^−1^ common for each formulation corresponds with the asymmetric and symmetric stretching vibrations of CH_2_ and CH_3_ groups present in all samples [56].

### 3.2. Evaluation of the Curing Reaction and Dynamics

The crosslinking process of EPB-ELO and tricomponent systems based on EPB-ELO and thiols (2SH and 3SH) was examined through FTIR spectroscopy, and the curing characteristics were determined by monitoring the consumption of the characteristic bands involved in the curing reaction.

To improve clarity, only representative spectra are shown in Figure 2, Figure 3 and Figure 4, highlighting the most relevant characteristic bands associated with epoxy, benzoxazine, thiol, and hydroxyl groups. This approach emphasizes the evolution of functional groups most indicative of the curing mechanism, while the complete series of stacked spectra is provided in the Supplementary Material (Figures S2–S10) for reference.

As expected, along with the increase in reaction time, the intensity of the signal representative of the benzoxazine (915 cm^−1^) and epoxy ring (840 cm^−1^) decreases in all samples. Concurrently, the appearance of a broad peak assigned to the –OH groups at ~3400 cm^−1^ indicates the successful ring-opening reactions [57,58].

Figure 4 shows the curing profile of the EPB-ELO system, highlighting the novel interactions developed between the components of the system. In this case, besides the thermal-activated benzoxazine ring-opening reaction and thermal-initiated homopolymerization of epoxy resin, the free amino groups can react simultaneously with both functionalities. For instance, one can observe that as the reaction proceeds, the signals associated with the amino groups (745, 1320, and 1464 cm^−1^) are consumed due to their high reactivity toward epoxy resin [59].

Apart from that, the phenolic hydroxyl groups formed during benzoxazine curing can react with the epoxy ring, forming novel ether linkage [60] that can be observed on the spectrum as a broad signal at 1080 cm^−1^. However, due to the presence of similar chemical bonds from the backbone of ELO, these newly formed interactions may overlap in the spectrum with the aforementioned ones. Despite that, their presence is confirmed by the different allures of the peak that become noticeable after 210 min of curing at 180 °C. This may suggest that the ether bond formation has a lower rate in comparison with the thermal polymerization. A possible explanation would be that due to a dense network formation in the first 3 h of polymerization, the mobility of reactive species is considerably reduced. Besides the phenolic hydroxyl, the tertiary amine from the Mannich bridge formed after benzoxazine cleavage can act as a catalyst to promote epoxy ring opening.

As curing proceeds, a strong signal appears on the spectra at 1660 cm^−1^, which is associated with C=O stretching from the amide group. Considering the curing conditions, the elevated temperatures may promote an amidation reaction between the ester [61] groups from the backbone of ELO and multiple amino groups prom PEI. Intriguingly, it can be observed that along with the increase in reaction extent, the signal characteristic to the allyl group from eugenol (1640 cm^−1^) gradually decreases, suggesting that the C=C bond may participate in the crosslinking process.

**Figure 3:**
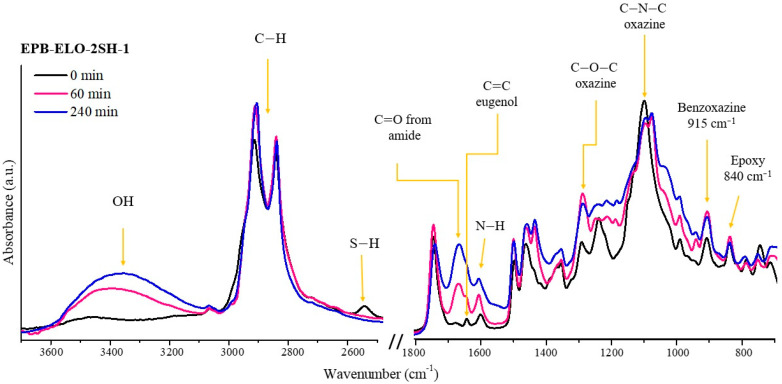
Time-dependent curing behavior for the EPB-ELO-2SH-1 system monitored through FTIR at selected curing times. The complete set of spectra is available in the Supplementary Material (Figure S6).

**Figure 4:**
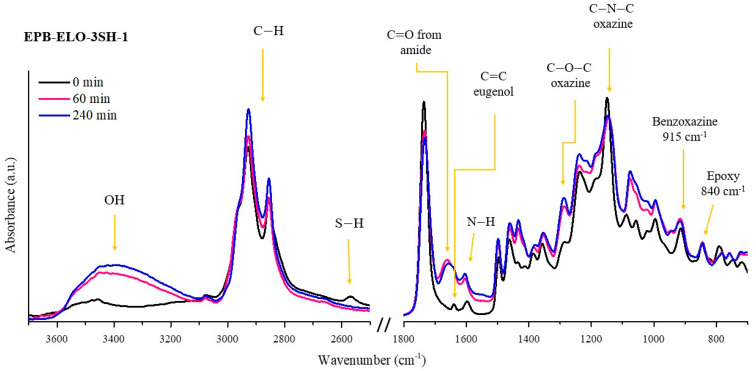
Time-dependent curing behavior for the EPB-ELO-3SH-1 system monitored through FTIR at selected curing times. The complete set of spectra is available in the Supplementary Material (Figure S10).

When thiols are introduced into the system, both their concentration and functionality influence the polymerization behavior and the resulting network structure. The structural modifications observed are illustrated in Figure 3 and Figures S3–S6 in the Supplementary Material for the 2SH blends, and in Figure 4 and Figures S7–S10 in the Supplementary Material for the 3SH blends. The spectra shown in Figure 3 and Figure 4 correspond to the formulations with the highest thiol concentration (1 g), while the complete datasets for all compositions are provided in the Supplementary Material (Figures S6 and S10).

The strong nucleophile character of the SH group makes it more susceptible to react with the epoxy moiety rather than benzoxazine due to its increased electrophilicity. In the thiol-epoxy/thiol-benzoxazine curing sequence, the hybrid network develops gradually as a consequence of the multiple species involved [62,63]. Thus, the amino groups from the benzoxazine oligomer do not only act as ring-opening promotors but also as catalysts for the nucleophilic addition reaction between thiol and epoxy due to their basic character. The characteristic decrease of the SH stretching band at ~2570 cm^−1^ within the first 30 min of curing, indicative of its high reactivity, is illustrated in the Supplementary Figures S3–S10 [64].

The main chemical bonds formed during the crosslinking process initiated by this functional group consist in β-hydroxy thioether, which can be observed in the spectra of each composition studied at ~3400 cm^−1^ alongside –OH. These newly formed chemical bonds have the ability to undergo additional exchange processes, such as transesterification. Such interactions can develop between the OH generated by the thiol-initiated epoxy ring opening and the multiple ester groups present on the backbone of ELO monomers and 3SH crosslinker. The dynamic character of these bonds represents a significant asset for the newly developed networks, as they can significantly contribute to the development of reprocessable materials [65].

Thiol-ene click reaction is another possible process that can occur due to the presence of the allyl group of eugenol [66,67]. This method usually requires the presence of free radicals that, in our case, can easily be generated during thermal treatment (180–210 °C). As stated above, the peak assigned to the C=C bond from eugenol exhibits a different consumption rate as a function of thiol functionality and concentration. This trend is more clearly evidenced in the Supplementary Figures S2–S10. Therefore, in the case of the ELO-ELO-3SH-1 sample, the allyl group is consumed after 30 min, while in the case of ELO-ELO-2SH-1 after 120 min as a consequence of the additional thiol moiety.

When it comes to the curing mechanism of conventional benzoxazine and epoxy resin, they follow relatively simple steps with a limited number of possible interactions. However, when they are combined, polymerization becomes more complex due to the formation of new reactive species during benzoxazine curing, such as phenolic -OH and tertiary amines coming from the Mannich bridge [68], that can further interact with the epoxy ring, promoting its cleavage.

In the case of EPB-ELO systems crosslinked by thiols, beside the oxazine and oxirane ring, thiols and numerous amine groups are present. It was previously demonstrated that these functional groups have a catalytic effect over both ring-opening reactions [69,70,71].

**Figure 5:**
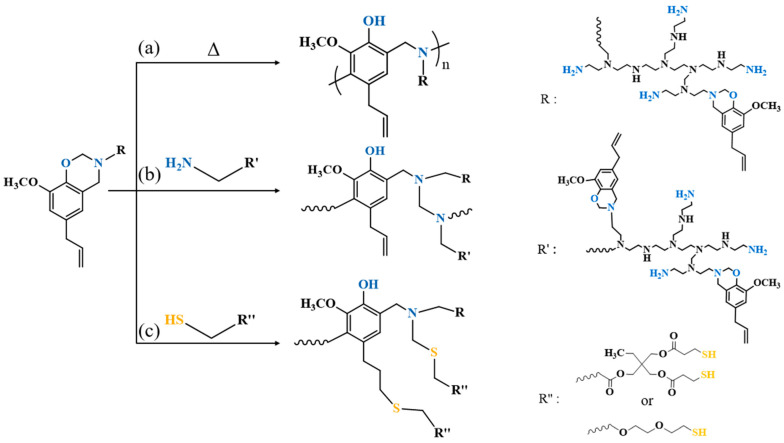
Possible reaction mechanisms for benzoxazine ring opening catalyzed by (**a**) temperature, (**b**) primary amine, and (**c**) thiols.

**Figure 6:**
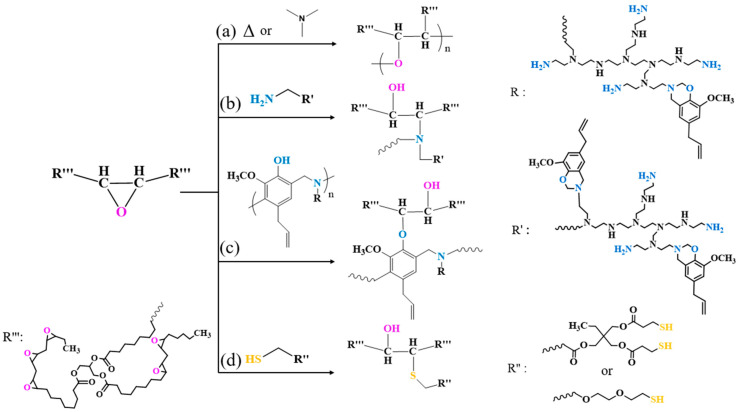
Schematic representation of possible epoxy ring-opening reactions catalyzed by (**a**) temperature or tertiary amines coming from the Mannich Bridge of polybenzoxazine or polyethyleneimine backbone, (**b**) primary amine, (**c**) phenolic OH of polybenzoxazine ring, and (**d**) thiols.

Therefore, as illustrated in Figure 5 and Figure 6, we propose seven potential reaction pathways for the studied systems, formulated in accordance with similar mechanisms reported in the literature [69,70,71].

It should be noted that, given the multicomponent nature of the investigated systems, the mechanistic interpretations presented here rely on correlations with the literature rather than direct validation using simplified model compounds with isolated functionalities. While such model studies would undoubtedly provide additional clarity, they are beyond the scope of the present work. Instead, our discussion builds on well-established curing behaviors of benzoxazines, epoxies, thiol-ene reactions, and amine-catalyzed processes [45,47,51,62,63], which together provide a consistent framework to rationalize the observed spectral evolutions. Thus, the proposed parallel curing pathways should be regarded as plausible hypotheses supported by both our experimental data and prior reports, rather than as conclusive evidence of isolated reaction mechanisms.

Analyzing the curing profile of the EPB-ELO system through FTIR (Figure S11), one can observe that there is a significant difference between the reaction conversion of benzoxazine over epoxy. Therefore, after 30 min of thermal curing at 180 °C, oxazine ring opening reaches ~40% conversion, while epoxy 10%. We can assume that in this case, the free amino groups that are closer to the oxazine ring catalyze this reaction, favoring the network formation. However, after 30 min of reaction, Mannich bridges and phenolic hydroxyl moieties are generated, and thus the epoxy ring-opening conversion starts to considerably increase due to the catalytic activity of the newly formed reactive species.

The allure of the curves describing the two polymerization processes depicts that benzoxazine curing develops in two main steps, while the epoxy network formation takes place in three stages. The additional step present in the case of the latter functionality is due to the existence of an induction stage, where the reactive species begin to form, and they can be observed on the graph in the first 50 min of reaction. After 4 h of curing at 180 °C, both components convert into a network up to 85–95% as a consequence of the autocatalytic nature of the process and reach full conversion after additional curing at 200 °C and 210 °C, which correspond with the final step of the reaction when conversion growth reaches a plateau. The slower rate of this final process is due to the increased viscosity of the system that limits the mobility of the remaining reactive sites, which now can form an additional crosslinking point in a diffusion-controlled manner.

The implications of the use of thiol-based crosslinkers (2SH and 3SH) were also evaluated by means of reaction progress over time studies, and the corresponding evolution of reaction extent over time is represented in Supplementary Figures S12 and S13. Analyzing the conversion graphs, it can clearly be observed that the induction period is common for all systems in the early stage of thermal treatment. The presence of multiple reactive functionalities within the system may lead to concurrence between epoxy and benzoxazine polymerization, as multiple latent catalytic species are generated. At the same time, it is widely known that in the case of polybenzoxazine formation, extensive hydrogen bonding is present within the system, which may induce a temporary stabilization, thus decreasing the reaction rate [72].

In can be observed clearly that while the concentration of the thiol crosslinker increases within the studied formulations, the overall reaction rate for both processes decreases in comparison with the EPB-ELO system (Supplementary Figures S14 and S15). This may be due to the fact that more reactive species will lead to an increase in the viscosity as well as crosslinking density, restricting the mobility of the molecular chains. However, the epoxy ring-opening reaction gradually rises due to the high reactivity of SH groups toward this functionality. When higher concentrations were used (0.5 and 1) for both 2SH and 3SH systems, the epoxy conversion surpassed the benzoxazine, demonstrating the high affinity of the radical species for this process. At the same time, the conversion of benzoxazine moiety showed a decline as a function of thiol concentration within the systems, suggesting that in this case, the free amines from its structure and the other reactive species (Mannich bridge and phenolic hydroxyl) are prone to activate the SH groups, promoting the epoxy crosslinking process.

**Scheme 1:**
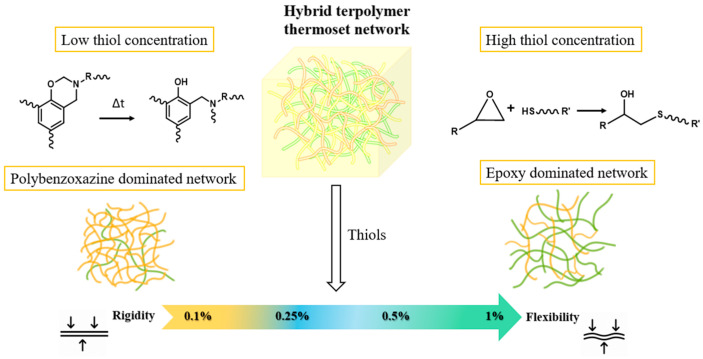
Schematic representation of the influence of thiol concentration on curing pathways and material properties.

According to the reaction progress over time data, we may conclude that in the case of the samples where thiols were used in lower concentrations (0.1 and 0.25), the polybenzoxazine networks develop first, while in the case of the samples with higher concentrations (0.5 and 1), the epoxy-based network prevails. Thereby, it is possible in the first case to obtain stiffer materials, whereas in the second case flexibility may be achieved as a consequence of the long aliphatic chains of ELO. These findings are summarized in Scheme 1, which illustrates the influence of thiol concentration on the dominant curing pathway and the resulting material properties.

### 3.3. Thermal Curing Behavior and Kinetics Monitored Through DSC

**Figure 7:**
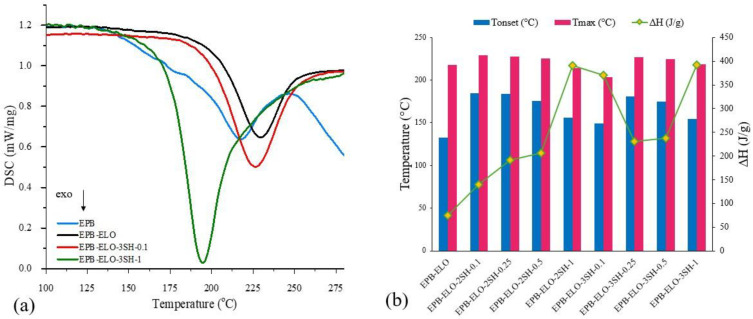
Representative DSC curves of EPB, EPB-ELO, and selected EPB-ELO3SH systems (**a**) and comparative analysis of DSC parameters (**b**) illustrating the effect of thiol concentration and functionality on curing behavior. For clarity, only representative samples are shown, while the complete set of DSC curves and data for all formulations is provided in the Supplementary Material (Figure S16 and Table S1).

DSC analysis was employed to determine the curing features of hybrid thermosets, with representative thermograms presented in Figure 7. Non-isothermal DSC analysis was employed to determine the curing activation energy for each system, and the corresponding results along with DSC parameters are presented in Table 2. The complete set of thermograms and extended tabulated data are available in the Supplementary Material (Figure S16 and Table S1).

The curve describing the curing process of EPB resin shows a broad exothermal event at 217.5 °C associated with the ring-opening polymerization of benzoxazine monomer. The allure of this thermal event along with its low-onset temperature suggests that in this case, the polymerization process takes place in multiple steps as a consequence of the multiple amino functionalities from its backbone. Right after polymerization takes place, another thermal event occurs in the case of the EPB sample, which corresponds with the first stage of thermal degradation [27]. Thus, considering the narrow window between polymerization and degradation, the use of polybenzoxazine by itself is limited due to the fact that it is more prone to degradation. As expected, in the case of ELO monomer, no polymerization event occurred since no curing agent was added. Over the last years, the copolymerization between benzoxazine and epoxy monomers was thoroughly investigated from the kinetic point of view, and the existing literature data [60,73] reported two distinct peaks on the DSC thermograms for such mixtures, representative of the curing process of each monomer. In the case of the EPB-ELO system, a single exothermal transition can be observed, suggesting a good compatibility between the two compounds and homogeneous network formation [74]. The allure of the peak along with the higher curing enthalpy (140 J/g) in comparison with EPB (70 J/g) demonstrates the catalytic effect of the amino groups [75] from the benzoxazine backbone, which in this case exerts a synergistic effect over both curing processes and promotes the chemical interactions among benzoxazine and epoxy, leading to a copolymer rather than an interpenetrated network. The increase in T_max_ associated with the polymerization of EPB-ELO, as compared to T_max_ of benzoxazine homopolymerization, as well as the highest value for activation energy (115 kJ/mol), support once again the existence of multiple chemical interactions that require more energy to take place.

The overall DSC data reveal a strong dependence between thiol composition and functionality over the curing parameters. Thus, as can be observed on both thermograms, the shape of the polymerization signal changes as a consequence of thiol type and concentration. The significant increase in enthalpy from 0.25 wt.% to 0.5 wt.% is attributed to a more favorable stoichiometric ratio between epoxy and thiol groups, which facilitates a more complete curing reaction and higher crosslinking density. This results in greater heat release during the reaction, especially in the case of 3SH, due to its higher functionality and ability to form a denser network.

The most notable difference may be noticed in the case of EPO-ELO-2SH-0.5 and EPB-ELO-3SH-0.5 samples, where apart from the broad allure of the peak, a shoulder appears at lower temperatures. This hidden step may be attributed to the epoxy-thiol reaction which, as demonstrated by the FTIR results for the reaction progress over time, becomes the dominant process after this concentration, exhibiting also an increased rate in comparison with the benzoxazine ring-opening process.

Moreover, along with the introduction of thiol within the EPB-ELO system, the T_max_ shifts to lower temperatures as both 2SH and 3SH concentration increases. This confirms the catalytic effect of thiols that have the ability not only to catalyze the benzoxazine ring opening but also to promote additional polymerization pathways, such as thiol-ene [76] and thiol-epoxy, lowering the energy barrier and leading to high crosslinking density. This effect is more prominent as the concentration increases and, thus, lower Ea values are obtained for the networks synthesized with 3SH as a consequence of the higher functionality of the crosslinker.

The multiple reaction sites and the numerous interactions that take place within the bio-based hybrid networks are dependent on the number of reactive species brought by the crosslinking agent, which grows as SH groups’ concentration increases [62]. Thus, curing enthalpy increases while activation energy considerably decreases as a function of thiol concentration.

The highest curing enthalpies were recorded for the samples with 0.5 and 1 wt.% 2SH and 3SH, respectively (370–410 J/g), which also had the lowest activation energies. This suggests that at higher thiol concentrations (0.5 and 1), a dense network can easily be obtained, releasing more heat upon formation. At the same time, the high reactivity of the SH groups impacts the activation energy, suggesting that for these systems the reactions are chemically controlled, and they occurred with a higher rate, while in the case of low concentrations (0.1 and 0.25 wt.%), a diffusion-controlled initiation takes place.

### 3.4. Thermal Stability and Thermomechanical Behavior

**Figure 8:**
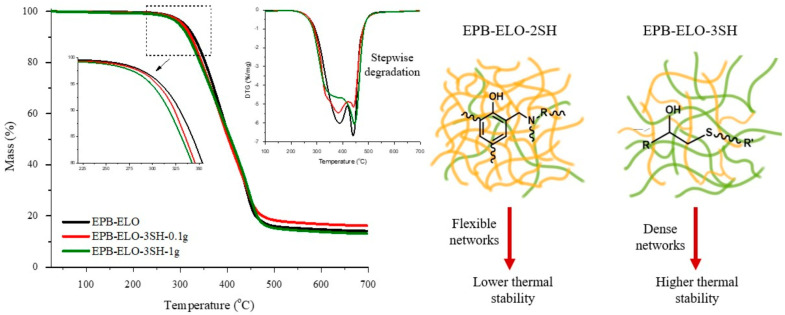
Representative TGA and DTG curves of EPB-ELO and EPB-ELO-3SH systems, highlighting the influence of thiol concentration and functionality on thermal stability. The complete sets of TGA and DTG curves for all compositions are provided in the Supplementary Material (Figures S17 and S18).

The effect of the incorporation of thiols as crosslinkers for the hybrid bio-based thermoset system comprising benzoxazine and epoxy was also assessed in terms of thermal properties. The TGA method was used to evaluate the influence of composition over thermal stability and degradation. Representative thermograms are shown in Figure 8, while the complete sets of data are provided in the Supplementary Material (Figures S17 and S18).

As can be observed by analyzing the thermal data presented in Table 3, the thiols do not significantly influence the thermal resistance of EPB-ELO coatings, all samples having the T_d3%_ around 300 °C. However, the slightly decreased values for thermal stability registered for the samples crosslinked with thiols as compared with EPB-ELO may be a consequence of the increased number of ether (C–O–C) and thio-ether bonds (C–S–C) formed during the crosslinking process [63]. It is well known that these chemical bonds are prone to thermal degradation up to 300 °C [77,78,79].

The residual mass after thermal decomposition is higher for the thiol-crosslinked samples in comparison with EPB-ELO thermoset, suggesting that a network with a higher crosslinking density will generate more char upon heating. Additionally, molecules that contain S and O atoms promote char formation, thus variations in this parameter can also be associated with the increase in thiol concentration and functionality [79].

Analyzing the DTG curves from Supplementary Figure S18, one may observe that in the case of EPB-ELO, the degradation takes place in two main steps. The DTG profiles show different degradation behaviors between 2SH- and 3SH-modified systems. This difference is attributed to the molecular structure and functionality of the thiols. The difunctional 2SH leads to a less densely crosslinked network, resulting in more complex, stepwise degradation, as seen by the appearance of secondary peaks or shoulders in the DTG curves. In contrast, 3SH, being tri-functional, promotes higher crosslinking density and network uniformity, leading to a more uniform and single-stage thermal degradation.

We may assume that according to FTIR reaction progress over time data, which suggest that benzoxazine networks develop faster than epoxy, in this case it is the first one to degrade at 388 °C as a consequence of the reduced thermal stability of EPB by itself. The next thermal event may correspond with the thermal decomposition of the ELO network at 440 °C. Along with the inclusion of thiols, the second decomposition step considerably decreases and becomes less prominent starting from the EPB-ELO-2SH-0.25 sample. In the case of EPB-ELO-3SH samples, the first step is the one that suffers alterations.

These events may be associated with the homogeneity of the networks, suggesting that after specific concentrations of thiols, a more equilibrated and compatible network can be obtained.

The residual mass was further employed to determine the limiting oxygen index with the aid of the Van Krevlen equation, and the corresponding results are presented in Table 3. All samples have values above 22, suggesting that they have good flame resistance.

**Figure 9:**
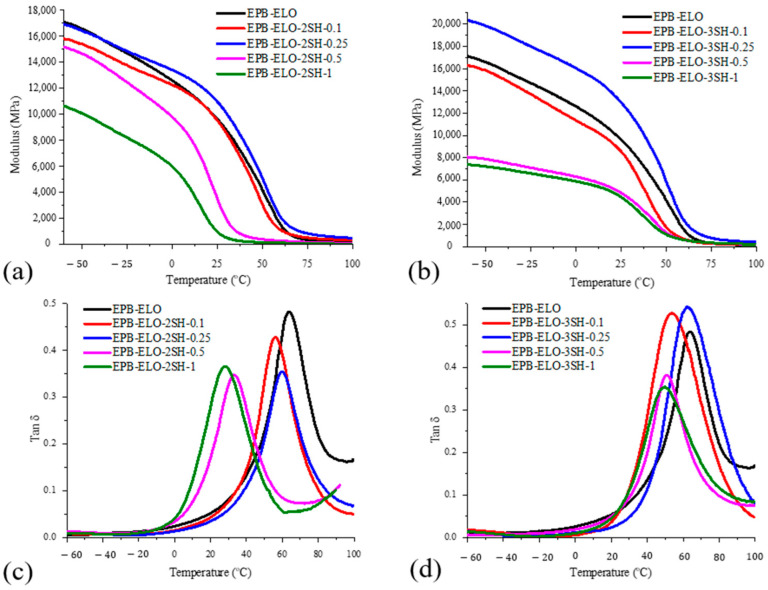
Dynamic mechanical analysis of hybrid thermosets based on EPB, ELO, and thiols: storage modulus (**a**,**b**) and tan δ (**c**,**d**) for 2SH and 3SH systems, respectively.

The viscoelastic features of the hybrid thermosets were evaluated by means of DMA, and the corresponding results are presented in Figure 9, while network features are displayed in Table 4. Storage modulus, a parameter associated with the stiffness of the material, shows a different conduct as a consequence of both thiol type and concentration. Due to the aliphatic nature of the SH-containing moieties, it is expected that they might behave as flexibilizing agents, reducing the rigidity of the system. This effect was noticed for all samples where thiols were used except for 0.25 wt.%-containing samples, where higher values for modulus were recorded. Analyzing the thermomechanical parameters presented in Table 4, one may observe that these systems have the highest crosslinking densities (4.3 and 4.12), which are almost twice as high compared with EPB-ELO material. These results suggest that 0.25 wt.%. thiol may represent the optimal concentration at which mechanical properties can be improved by achieving good interfacial interaction and synergy between all the reactive species. Even though the crosslinking density of the EPB-ELO-3SH-0.25 is lower than EPB-ELO-2SH-0.25, it has the highest storage modulus. This is a consequence of the functionality of the thiol, which due to the branched structure may induce rigidity and consequently additional intermolecular interactions, such as hydrogen bonding.

The networks containing 0.5 and 1% 2SH and 3SH display reduced stiffness as a result of the increased concentration of thiols. In this case, elastic behavior is dominant, as it is also suggested by the reduced values for Tg (<50 °C), which are characteristic for elastomeric materials.

The allure of the tan δ peak gives valuable insights about the structure of the network. As it can be observed from Figure 9c,d, the existence of a single peak suggests good compatibility between the components of the systems. As seen from Table 4, the Tg of the hybrid networks decreases as thiol concentration increases, highlighting the plasticizing effect of the thiol moieties over the hybrid thermosets.

Molecular weight between crosslinking points (M_c_) is another key parameter with a strong impact over the Tg, which is inversely proportional with the crosslinking density. Thus, one may observe that along with the inclusion of thiols, different network arrangements or mesh sizes may be obtained as a consequence of their chemical structures. Thus, higher values determined for EPB-ELO and EPB-ELO-2SH-1 suggest a broad interchained spacing of the macromolecules, which consequently will lead to a higher free volume. On the contrary, the lowest values determined for EPB-ELO-2SH-0.25 and EPB-ELO-3SH-0.25 demonstrate a compact network structure.

Evaluation of the gel content of a crosslinked network gives valuable information about the efficiency of the curing process and network stability. The higher values for gel content determined by all studied systems suggest that dense and stable networks were obtained, having small fractions of residual monomers (~5%) [80].

According to thermomechanical data, the performance of the hybrid thermosets can be tailored to meet specific requirements for different applications. Achieving a good balance between high Tg and crosslinking density while maintaining a stiff and compact structure may be performed by adapting both the composition and type of thiol involved as a crosslinking agent.

### 3.5. Nanomechanical and Surface Properties

**Figure 10:**
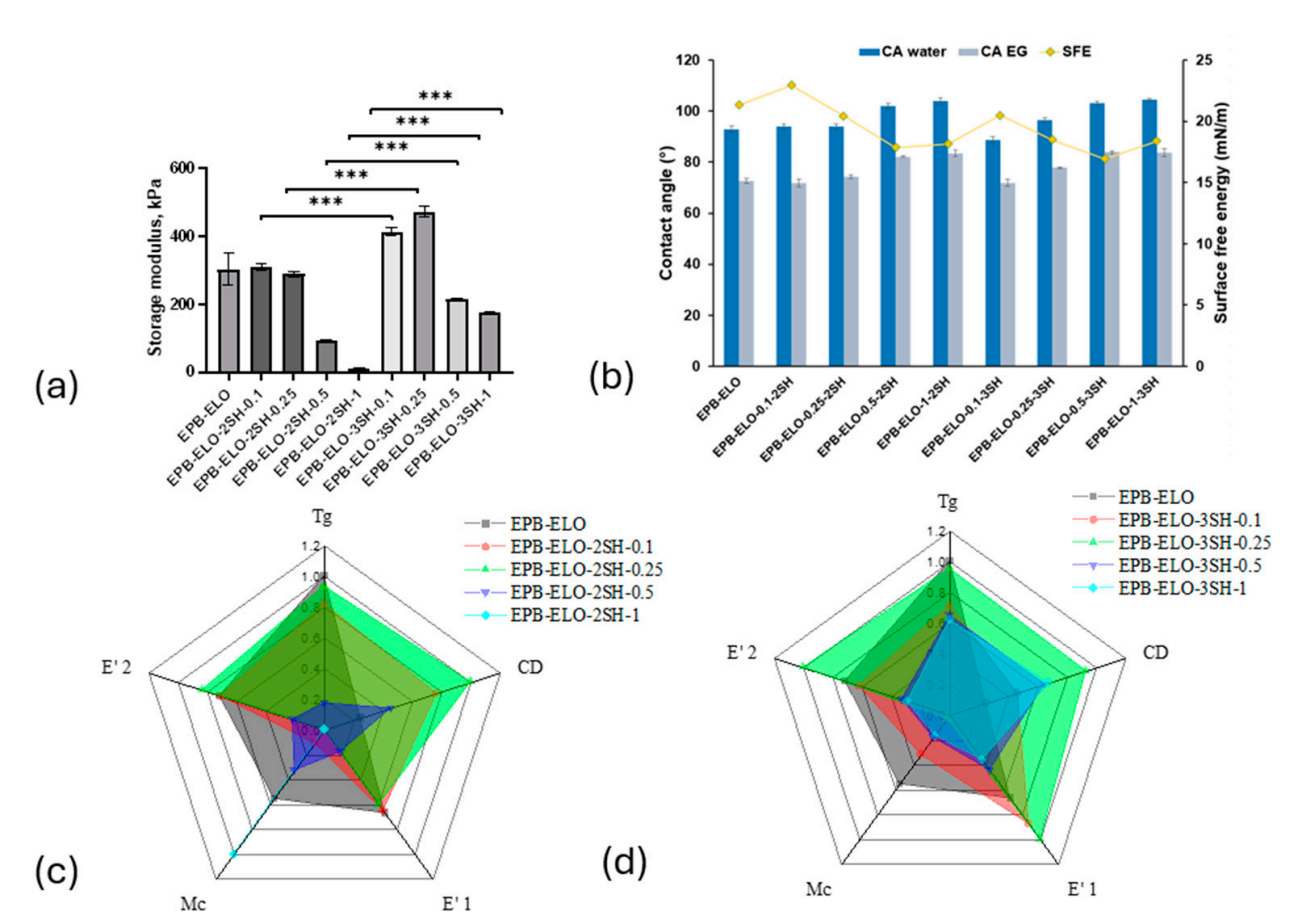
(**a**) Nanomechanical properties (of the synthesized hybrid thermosets based on EPB, ELO, and thiols). Statistical significance: ns—not significant, * *p* < 0.05, ** *p* < 0.01, and *** *p* < 0.001. (**b**) Surface properties of bio-based benzoxazine-epoxy thermosets crosslinked with thiols. Spider plots of mechanical parameters associated with the synthesized hybrid thermosets containing S2H (**c**) and 3SH (**d**). Tg—glass transition temperature from DMA; CD—crosslinking density computed from DMA data; Mc—molecular weight between crosslinking points computed from DMA data; E′_1_—storage modulus from nanoindentation results; E′_2_—storage modulus at 25 °C from DMA data.

Mechanical properties at the nanoscale were evaluated through nanoindentation, and the corresponding results are shown in Figure 10a. Amongst the formulations studied, the highest mechanical stiffness was measured for EPB-ELO-3SH-0.25 samples, while EPB-ELO-2SH-1 demonstrated the highest ductility. The difference in storage modulus trends between the indentation test and DMA results is due to the distinct nature of the two techniques. DMA measures the bulk viscoelastic properties, while nanoindentation captures localized, surface-sensitive mechanical behavior. The composition, particularly the type and content of thiol (2SH vs. 3SH), affects the crosslinking density and network homogeneity, which may vary between the bulk and surface regions. For instance, systems with higher 3SH content tend to form more densely crosslinked and uniform networks, reflected in both techniques. In contrast, 2SH-containing systems may show more heterogeneous structures, leading to discrepancies between surface and bulk measurements. Thus, trifunctional thiol contributes to the formation of multiple crosslinking points, while difunctional thiol acts as a chain extender or is plasticized within the hybrid network.

Considering the main application for the synthesized thermoset as coating materials, the evaluation of the surface properties represents a valuable technique, as they strongly influence adhesion, functionality, and performance. The contact angle results and surface-free energy for the hybrid thermosets are presented in Figure 10b.

From a structural point of view, the EPB-ELO sample includes both numerous polar groups, such as amino, and nonpolar moieties coming from the long aliphatic chains of epoxidized oils and exhibits a water contact angle of 92°, suggesting slightly hydrophobic features. Apart from the initial composition, numerous polar OH groups are generated during both benzoxazine and epoxy polymerization, which should normally lead to increased water affinity. Nevertheless, during the curing reaction, extensive hydrogen bonding occurs within the matrix and influences the wettability and surface properties of all samples, shifting the contact angle values to hydrophobicity.

The use of thiol-based crosslinkers has a significant influence over surface properties of hybrid thermosets. As the concentration increases, the water affinity gradually decreases, reaching maximum values for the samples with the highest thiol concentrations. Thus, EPB-ELO-2SH-1 has a contact angle value of 103.9°, while EPB-ELO-3SH-1 has a value of 104.5°.

These results are in agreement with the crosslinking density computed from DMA results (Figure 10c,d), suggesting that compact networks are obtained with strong intermolecular hydrogen bonding within the formulations.

### 3.6. Hydrolytic Stability and Degradation of Hybrid Thermosets

During the crosslinking process of the hybrid thermosets, numerous ester bonds, such as β-hydroxy ester and β-thioester, are formed between the components. It is already known that the presence of these functionalities within thermoset materials improves adhesion and flexibility due to their polar nature and ability to interact with other moieties. Apart from that, one of the most interesting features is their sensitivity to hydrolysis reactions, which can be exploited to develop materials with tunable degradability.

**Figure 11:**
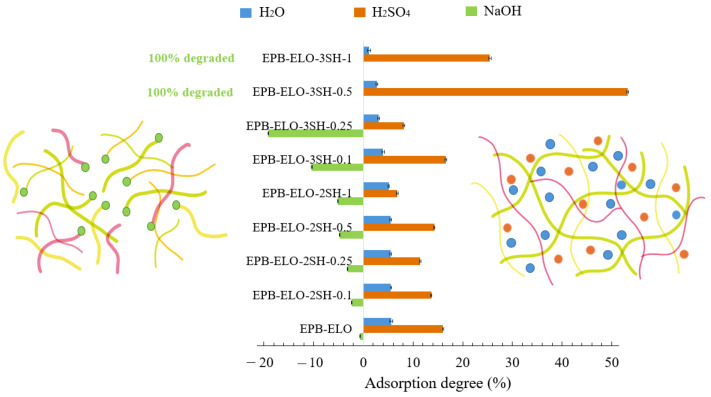
Adsorption profiles for terpolymer thermosets based on EPB, ELO, and thiols.

The ester group hydrolysis was evaluated by adsorption tests under basic (NaOH 1 M) and acid (H_2_SO_4_ 1 M) conditions, and the corresponding results are presented in Figure 11. Although all samples exhibited slight absorption when immersed in water, there was a slight decrease in the case of EPB-ELO crosslinked with 3SH. In this case, these materials may be characterized by a more stable internal hydrogen bonding network that restricts the mobility of the water molecules.

When exposed to basic environment, thiol-based polymeric networks started to gradually degrade until complete decomposition in the case of EPB-ELO-3SH-0.1 and EPB-ELO-3SH-1 samples. These findings suggest that the thermosets are more prone to nucleophilic attack in alkaline conditions, leading to complete disruption of the structural integrity of the materials [80]. Apart from that, the gradual increase in decomposition as thiol type and concentration increase may be an indicator for the content of ester groups, which are strongly related to the SH moieties.

Interestingly, when exposed to acidic conditions, the adsorption capacity increased significantly. This may be caused by the ability of H_2_SO_4_ to protonate sulfur-containing functionalities, such as thioesters, increasing polarity and consequently the adsorption capability. At the same time, the internal hydrogen bonds network may be disrupted, and different internal rearrangement of the macromolecular chains can be promoted, creating more space within the network.

## 4. Conclusions

A series of novel bio-based ternary thermosets were successfully synthesized by employing thiols as curing agents for hybrid bio-based epoxy/benzoxazine monomers. The effects of thiols on copolymerization behavior, thermal stability, and mechanical properties were thoroughly investigated.

This study demonstrated that amine-functional EPB resins react with ELO and thiol crosslinkers to form hybrid thermosets with tunable curing behavior, network structure, and properties. FTIR and DSC analyses revealed that thiol concentration and functionality strongly influenced polymerization kinetics: higher thiol levels accelerated epoxy ring opening, shifted curing to lower temperatures, and promoted dense network formation, while lower concentrations favored benzoxazine polymerization. TGA results showed that thiol incorporation did not significantly influence thermal stability, though minor reductions in resistance were linked to ether and thio-ether bonds. DMA measurements indicated an optimal thiol content (~0.25 wt.%) for maximizing modulus and crosslinking density, whereas higher levels produced more elastomeric materials with reduced Tg. Surface characterization confirmed improved hydrophobicity with increasing thiol content, but basic conditions accelerated degradation due to ester hydrolysis. Overall, thiol-modified EPB-ELO thermosets offer a versatile platform for tailoring thermal, mechanical, and surface properties through controlled composition and crosslinking chemistry.

## Figures and Tables

**Figure 2:**
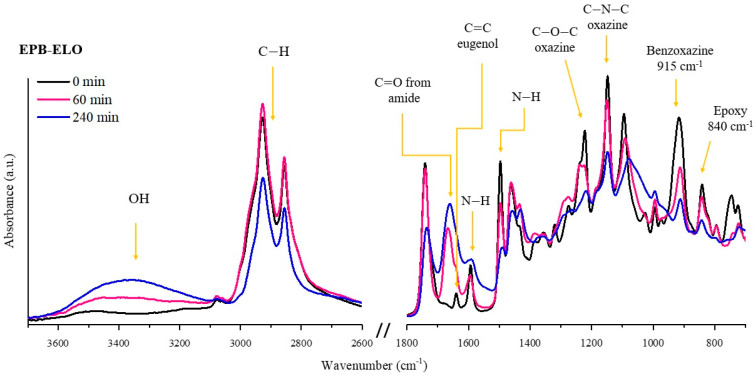
Time-dependent curing behavior for the EPB-ELO system monitored through FTIR at selected curing times. The complete set of spectra is available in the Supplementary Material (Figure S2).

**Table 1:** Compositions and abbreviations for the synthesized hybrid networks.

Sample Name	Sample Composition			
	Benzoxazine	Epoxy	Thiol	Mass Ratio Between the Components (wt.%)
EPB-ELO	EPB	ELO	-	1:1:0
EPB-ELO-2SH-0.1	EPB	ELO	2SH	1:1:0.1
EPB-ELO-2SH-0.25	EPB	ELO	2SH	1:1:0.25
EPB-ELO-2SH-0.5	EPB	ELO	2SH	1:1:0.5
EPB-ELO-2SH-1	EPB	ELO	2SH	1:1:1
EPB-ELO-3SH-0.1	EPB	ELO	3SH	1:1:0.1
EPB-ELO-3SH-0.25	EPB	ELO	3SH	1:1:0.25
EPB-ELO-3SH-0.5	EPB	ELO	3SH	1:1:0.5
EPB-ELO-3SH-1	EPB	ELO	3SH	1:1:1

**Table 2:** Activation energy values obtained from DSC data for bio-based ternary networks.

Sample	Ea Kissinger (kJ/mol)	Ea Ozawa (kJ/mol)	Ea Average (kJ/mol)
ELO	-	-	-
EPB	-	-	-
EPB-ELO	118.3	111.1	114.7
EPB-ELO-2SH-0.1	95.6	99.1	97.3
EPB-ELO-2SH-0.25	91.3	94.8	93.1
EPB-ELO-2SH-0.5	82.7	86.6	84.6
EPB-ELO-2SH-1	53.2	58.5	55.9
EPB-ELO-3SH-0.1	96.6	99.9	98.2
EPB-ELO-3SH-0.25	95.9	99.2	97.5
EPB-ELO-3SH-0.5	89.5	93.1	91.3
EPB-ELO-3SH-1	72.2	76.3	76.8

**Table 3:** TGA data obtained for bio-based hybrid networks.

Sample	T_d5%_ (°C)	T_d10%_ (°C)	T_max_ (°C)		Residual Mass (%)	LOI (%)
EPB-ELO	309.2	329.3	387.6	440.3	14.1	23.1
EPB-ELO-2SH-0.1	304.2	323.5	375.1	438.5	14.7	23.4
EPB-ELO-2SH-0.25	306.2	326.2	380.5		22.1	26.3
EPB-ELO-2SH-0.5	308.7	325.4	365.3		18.1	24.7
EPB-ELO-2SH-1	290.5	311.3	387.5		19.4	25.3
EPB-ELO-3SH-0.1	304.9	323.6	382.1	441.2	16.2	24.0
EPB-ELO-3SH-0.25	306.4	325.7	388.1	442.1	18.0	24.7
EPB-ELO-3SH-0.5	304.2	322.7	381.4	438.2	15.4	23.7
EPB-ELO-3SH-1	298.6	317.7	350.1	445.8	13.1	22.7

**Table 4:** Network properties for hybrid terpolymeric thermosets.

Sample	E′ at 25 °C (MPa)	Tg (°C)	CD × 10^3^ (mol/cm^3^)	M_c_ (g/mol)	Gel Fraction (%)
EPB-ELO	11,865.9	62.6	2.38	1524.4	99.1
EPB-ELO-2SH-0.1	9422.6	56.2	3.71	975.8	94.7
EPB-ELO-2SH-0.25	10,855.6	60.2	4.30	843.5	96.9
EPB-ELO-2SH-0.5	3505.2	33	2.91	1245.4	93.7
EPB-ELO-2SH-1	886.6	26.8	1.75	2073.6	93.7
EPB-ELO-3SH-0.1	10,827.4	52.4	2.93	1234.4	94.2
EPB-ELO-3SH-0.25	9402.1	60.9	4.12	880.3	94.5
EPB-ELO-3SH-0.5	3536.1	50	3.40	1064.7	94.3
EPB-ELO-3SH-1	979.3	48.9	3.47	1044.8	99.7

CD—crosslinking density; M_c_—molecular weight between crosslinking points; E′—storage modulus.

## Data Availability

The raw data supporting the conclusions of this article will be made available by the authors on request.

## Supplementary Materials

The following supporting information can be downloaded at: https://www.mdpi.com/article/10.3390/polym17172389/s1. Figure S1: The chemical structures of the reactants used in the synthesis of hybrid networks. Figure S2: Time-dependent curing behavior of EPB-ELO system monitored for 240 min at 180 °C through FTIR. Figure S3: Time-dependent curing behavior of EPB-ELO-2SH-0.1 system monitored for 240 min at 180 °C through FTIR. Figure S4: Time-dependent curing behavior of EPB-ELO-2SH-0.25 system monitored for 240 min at 180 °C through FTIR. Figure S5: Time-dependent curing behavior of EPB-ELO-2SH-0.5 system monitored for 240 min at 180 °C through FTIR. Figure S6: Time-dependent curing behavior of EPB-ELO-2SH-1 system monitored for 240 min at 180 °C through FTIR. Figure S7: Time-dependent curing behavior of EPB-ELO-3SH-0.1 system monitored for 240 min at 180 °C through FTIR. Figure S8: Time-dependent curing behavior of EPB-ELO-3SH-0.25 system monitored for 240 min at 180 °C through FTIR. Figure S9: Time-dependent curing behavior of EPB-ELO-3SH-0.5 system monitored for 240 min at 180 °C through FTIR. Figure S10: Time-dependent curing behavior of EPB-ELO-3SH-1 system monitored for 240 min at 180 °C through FTIR. Figure S11: The reaction extent of EPB-ELO system determined from FTIR data. Figure S12: Reaction extent for EPB-ELO-2SH systems. Figure S13: Reaction extent for EPB-ELO-3SH systems. Figure S14: Comparative curing conversion of benzoxazine moiety as a function of thiol type and concentration. Figure S15: Comparative curing conversion of epoxy moiety as a function of thiol type and concentration. Figure S16: DSC thermogram of hybrid networks. Figure S17: TGA curves for hybrid networks. Figure S18: DTG curves for hybrid networks. Table S1: DSC parameters for bio-based ternary networks.
