# Supplementary material for: Multi-Functional Hybrid Terpolymer Thermosets Based on Thiols Bio-Based Epoxy and Benzoxazine Monomers
Source: Polymers (Basel). 2025 Sep 1;17(17):2389. doi: 10.3390/polym17172389 (PMC12431179; doi:10.3390/polym17172389)
Supplement: Supplementary file 1 [file polymers-17-02389-s001.zip › polymers-3794895-supplementary.docx]

Multi-functional hybrid terpolymer thermosets based on thiols bio-based epoxy and benzoxazine monomers

Madalina Ioana Necolau^1,2^, Elena Iuliana Biru^1,2^, Elena Olaret^1^, Horia Iovu^1,2^*

*^1^ Advanced Polymer Materials Group, National University of Science and Technology POLITEHNICA Bucharest, Romania*

*^2^ Academy of Romanian Scientists, Ilfov 3, 050044 Bucharest, Romania*

*Corresponding author email: horia.iovu@upb.ro


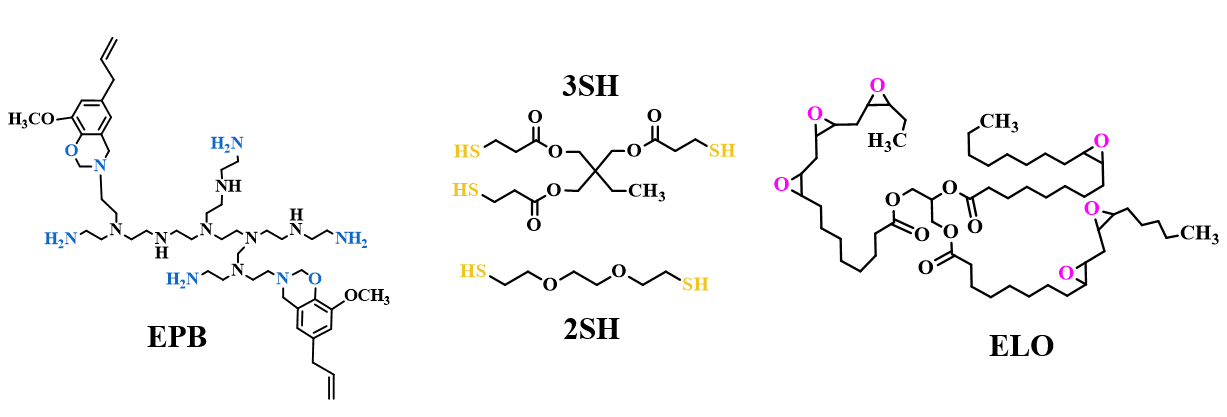


Figure S1. The chemical structures of the reactants used in the synthesis of hybrid networks


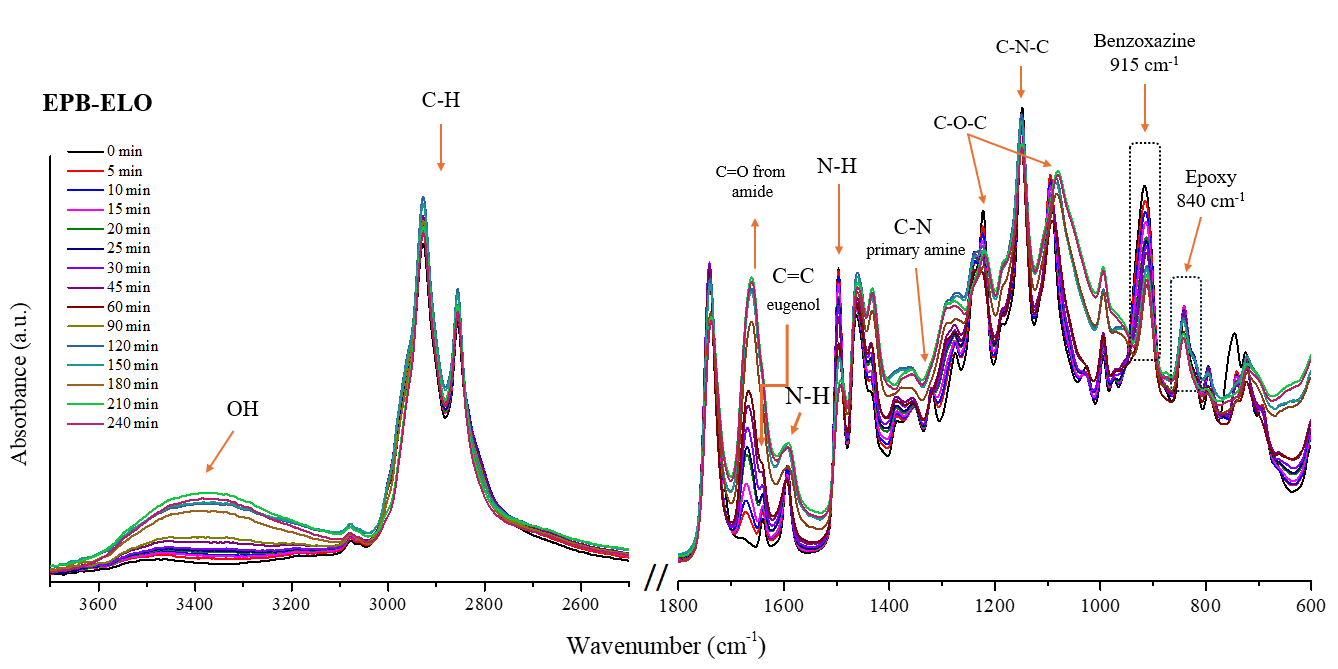
 Figure S2. Time-dependent curing behavior of EPB-ELO system monitored for 240 min at 180 °C through FTIR


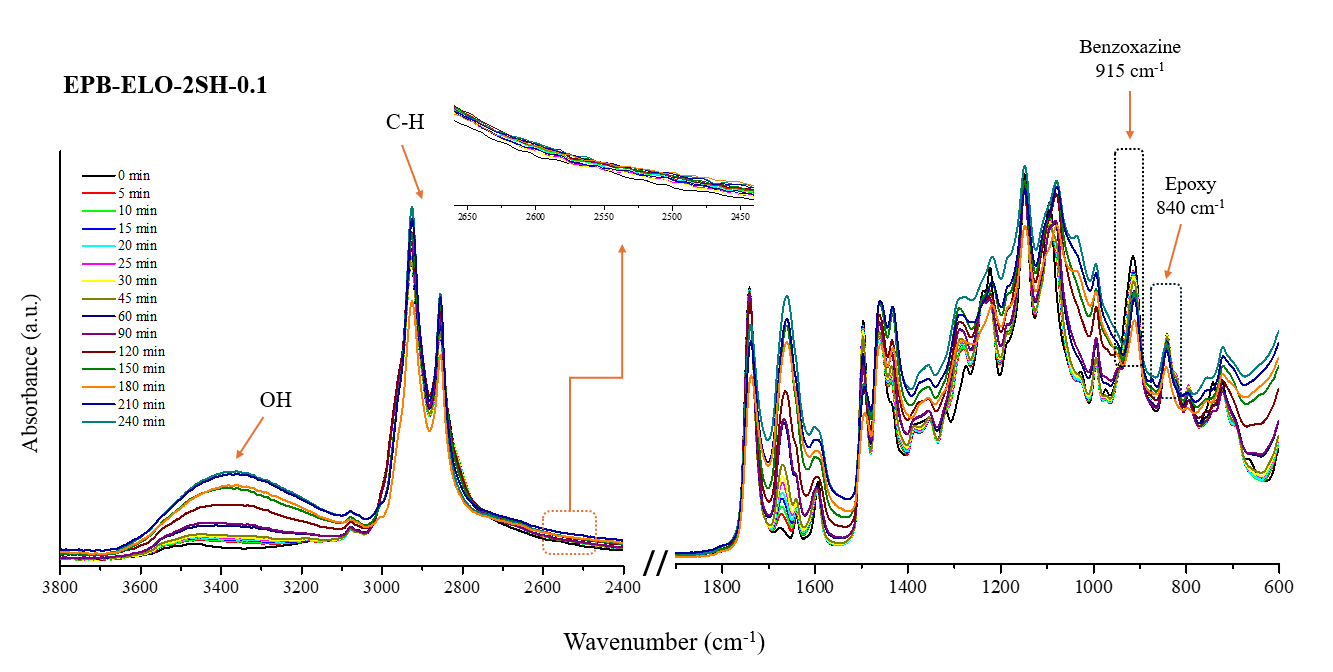


Figure S3. Time-dependent curing behavior of EPB-ELO-2SH-0.1 system monitored for 240 min at 180 °C through FTIR


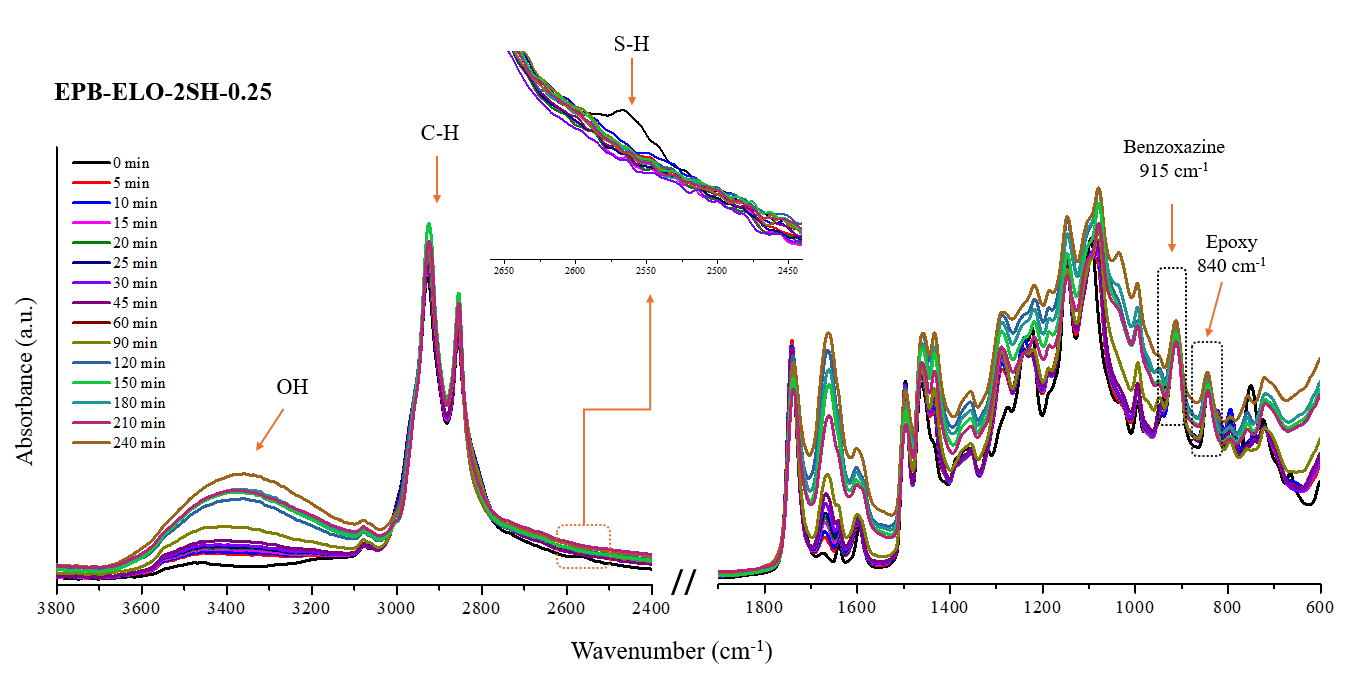


Figure S4. Time-dependent curing behavior of EPB-ELO-2SH-0.25 system monitored for 240 min at 180 °C through FTIR


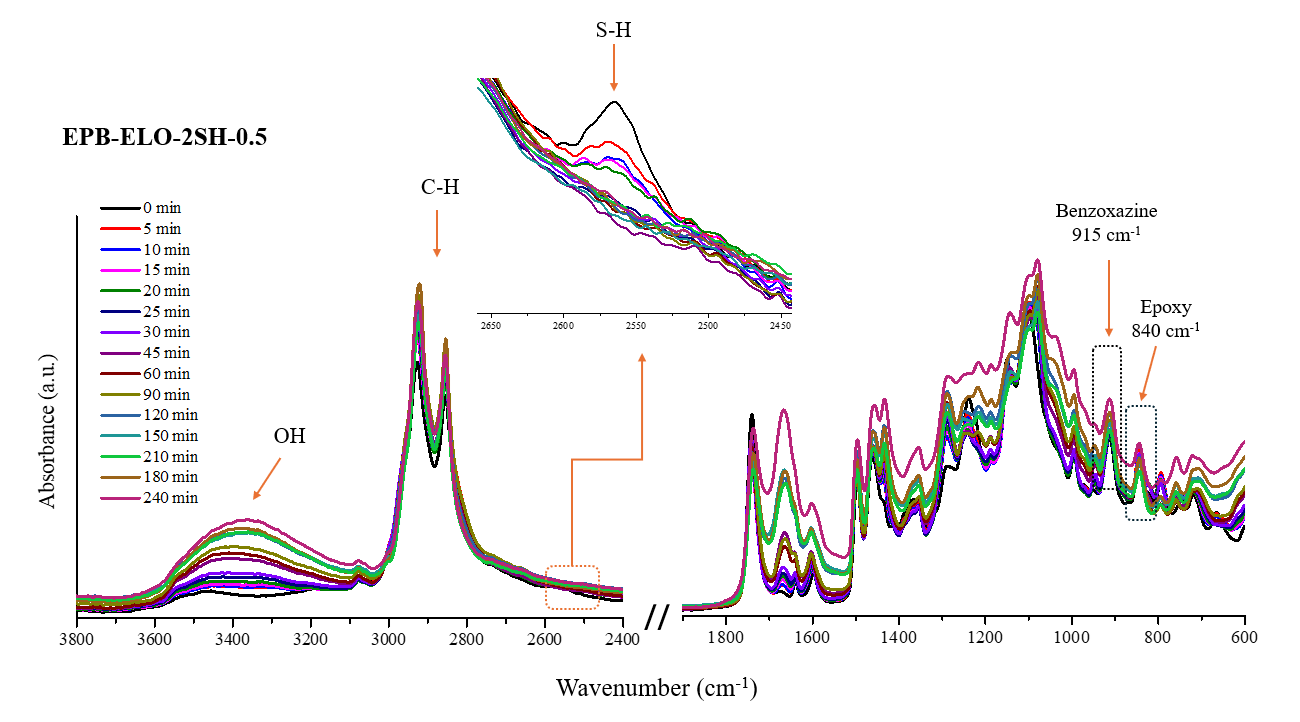


Figure S5. Time-dependent curing behavior of EPB-ELO-2SH-0.5 system monitored for 240 min at 180 °C through FTIR


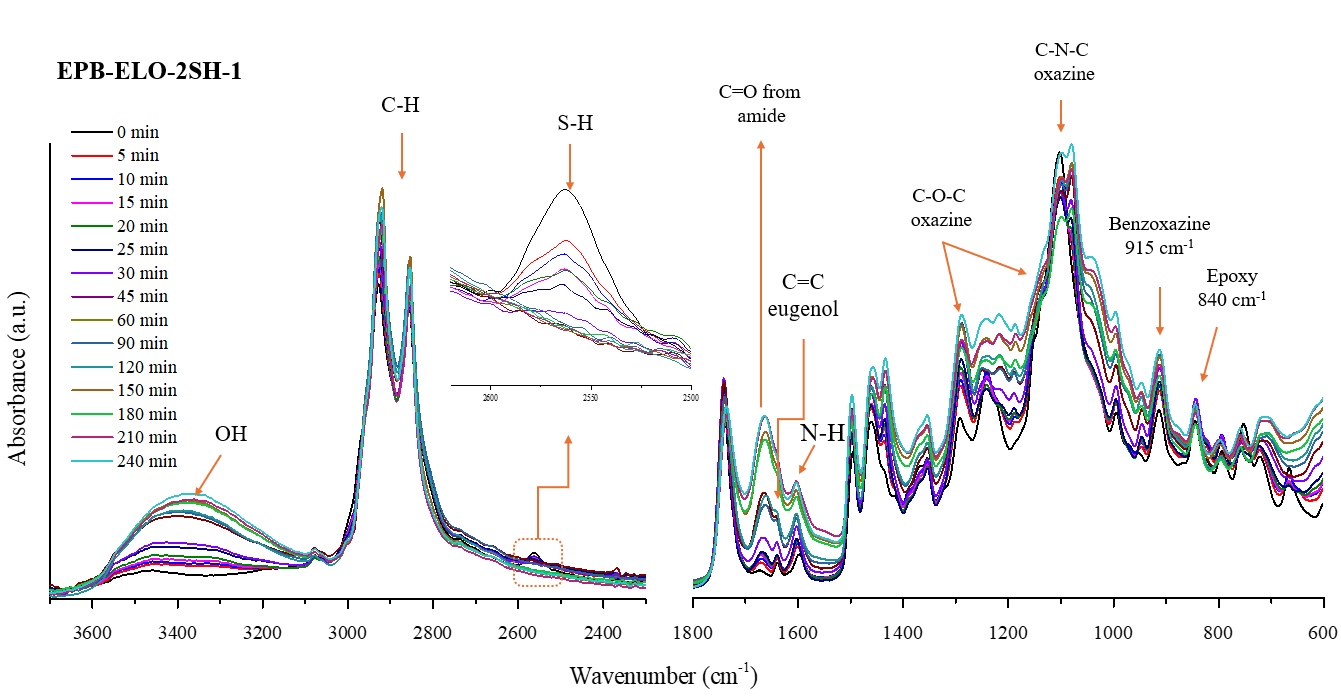


Figure S6. Time-dependent curing behavior of EPB-ELO-2SH-1 system monitored for 240 min at 180 °C through FTIR


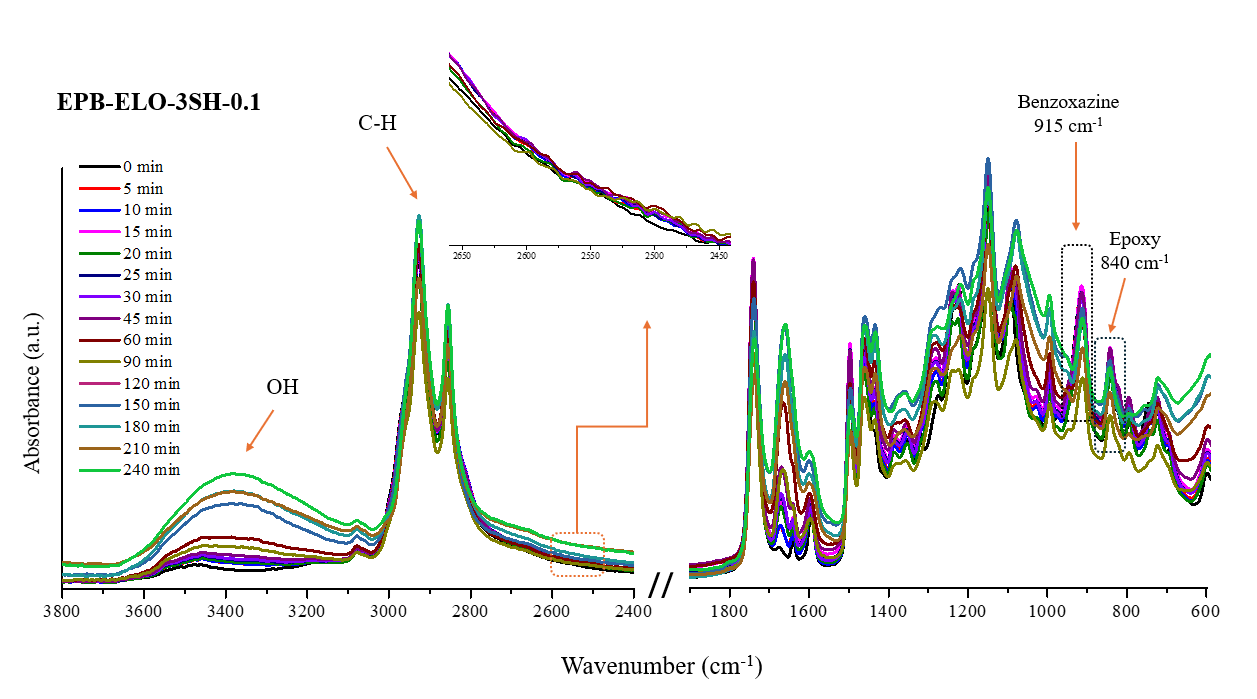


Figure S7. Time-dependent curing behavior of EPB-ELO-3SH-0.1 system monitored for 240 min at 180 °C through FTIR


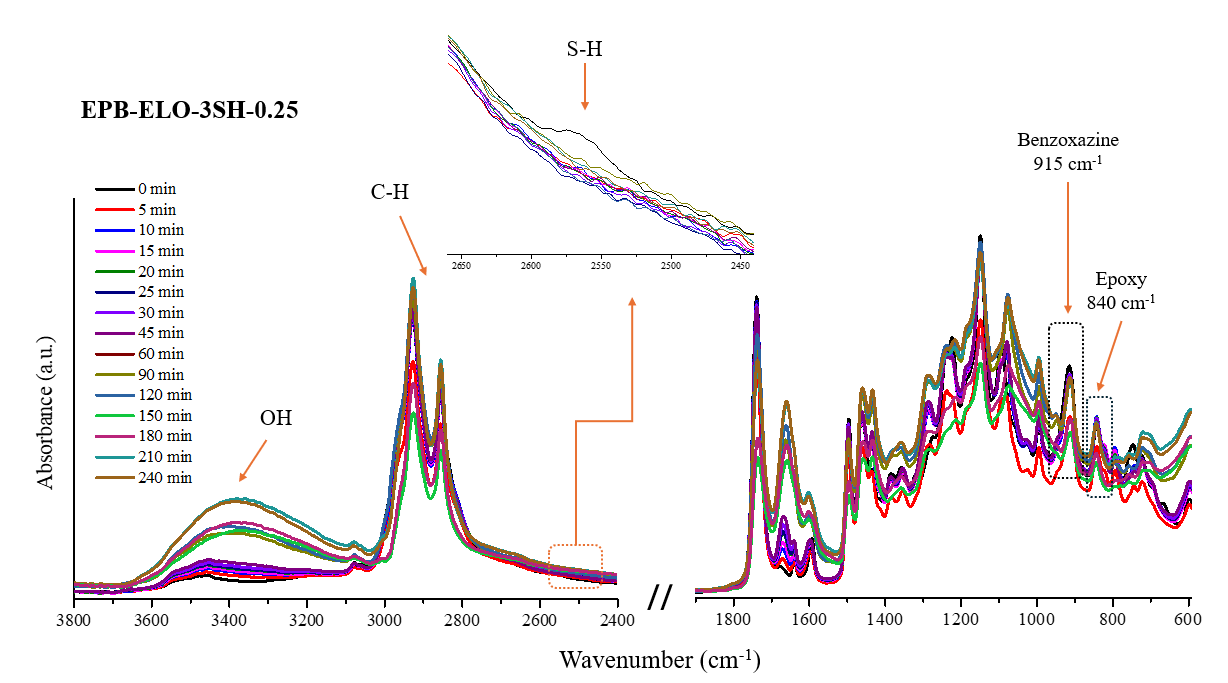


Figure S8. Time-dependent curing behavior of EPB-ELO-3SH-0.25 system monitored for 240 min at 180 °C through FTIR


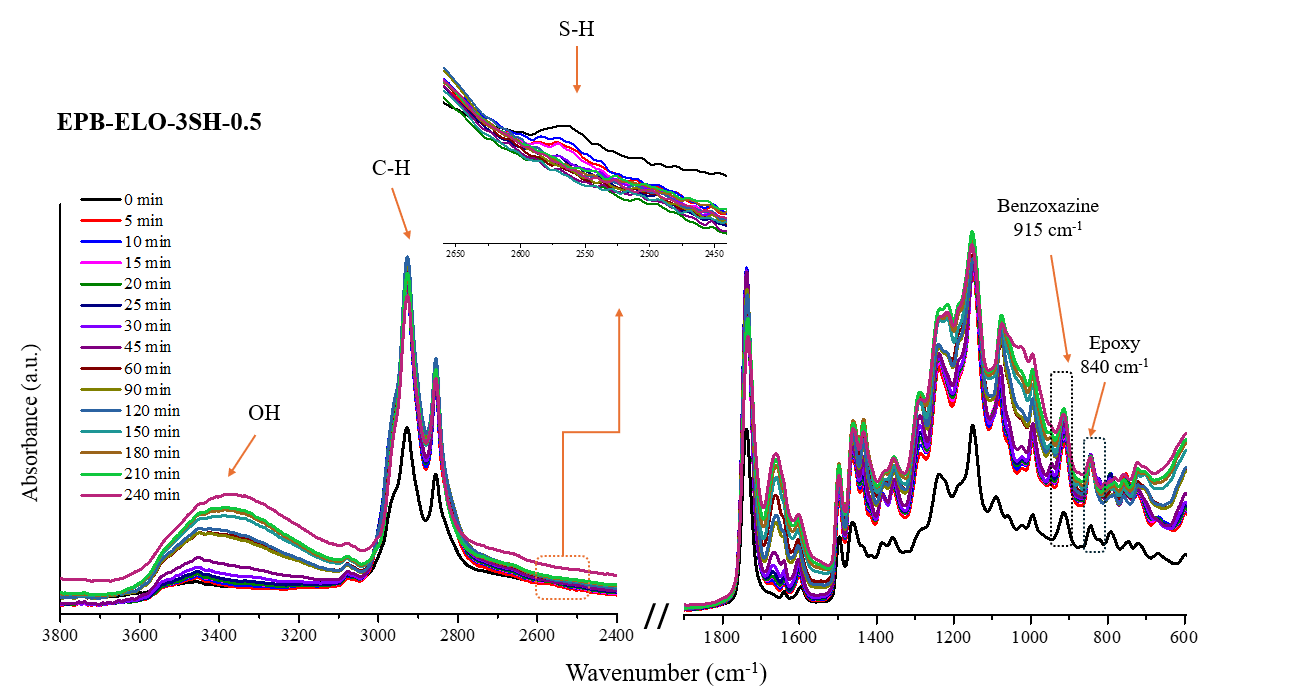


Figure S9. Time-dependent curing behavior of EPB-ELO-3SH-0.5 system monitored for 240 min at 180 °C through FTIR


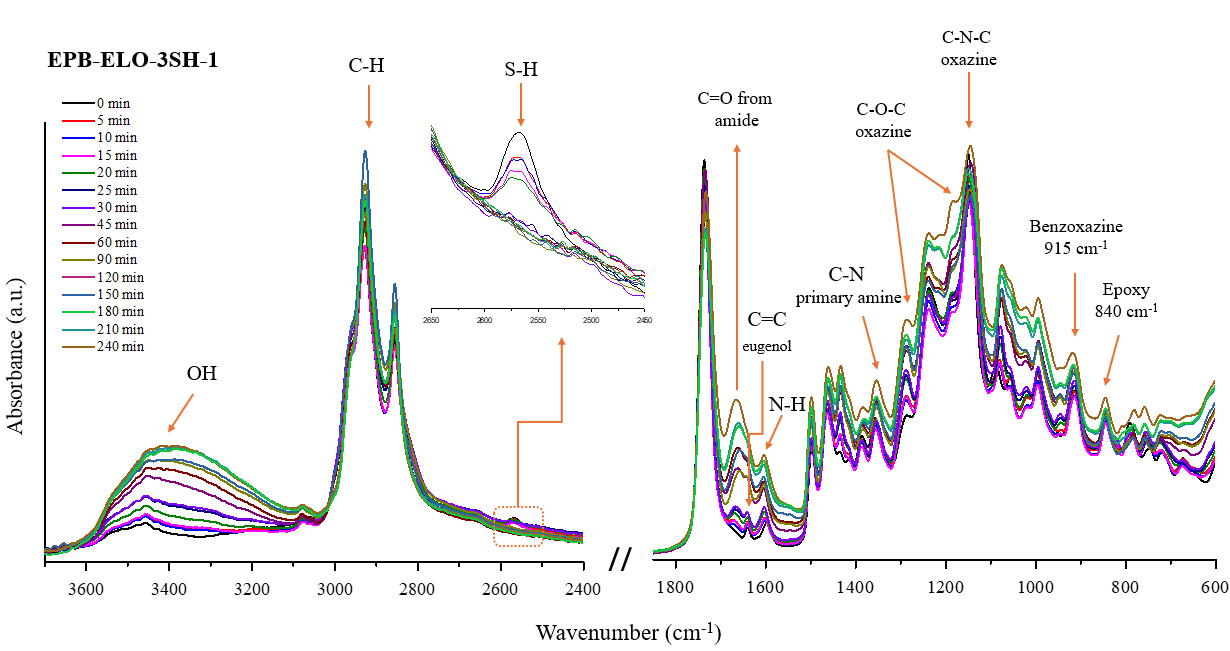


Figure S10. Time-dependent curing behavior of EPB-ELO-3SH-1 system monitored for 240 min at 180 °C through FTIR

Figure S11. The reaction extent of EPB-ELO system determined from FTIR data.


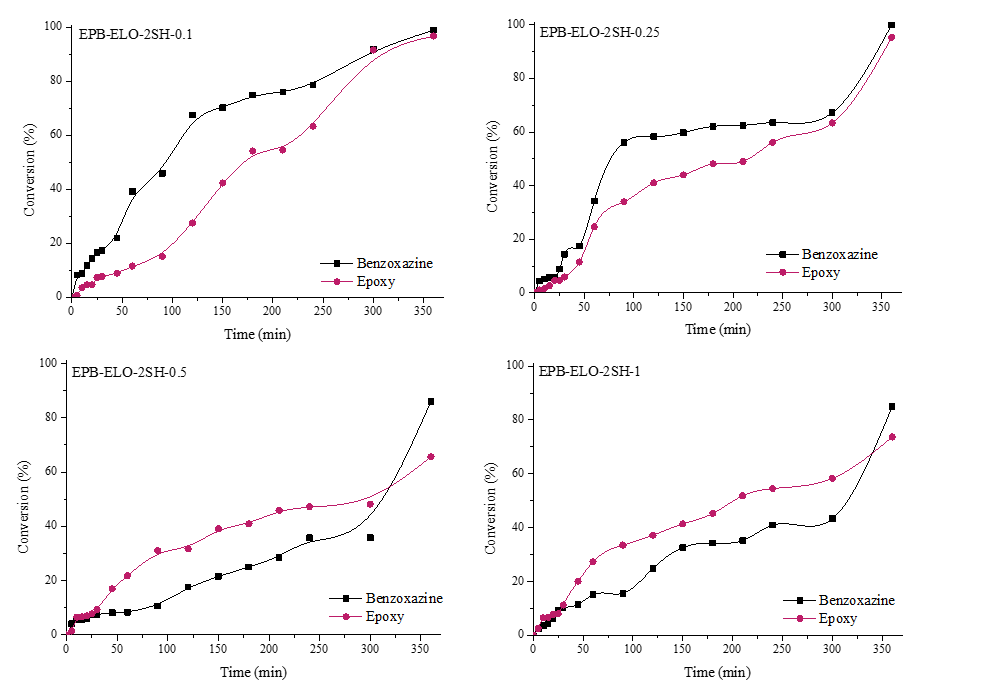


Figure S12. Reaction extent for EPB-ELO-2SH systems.


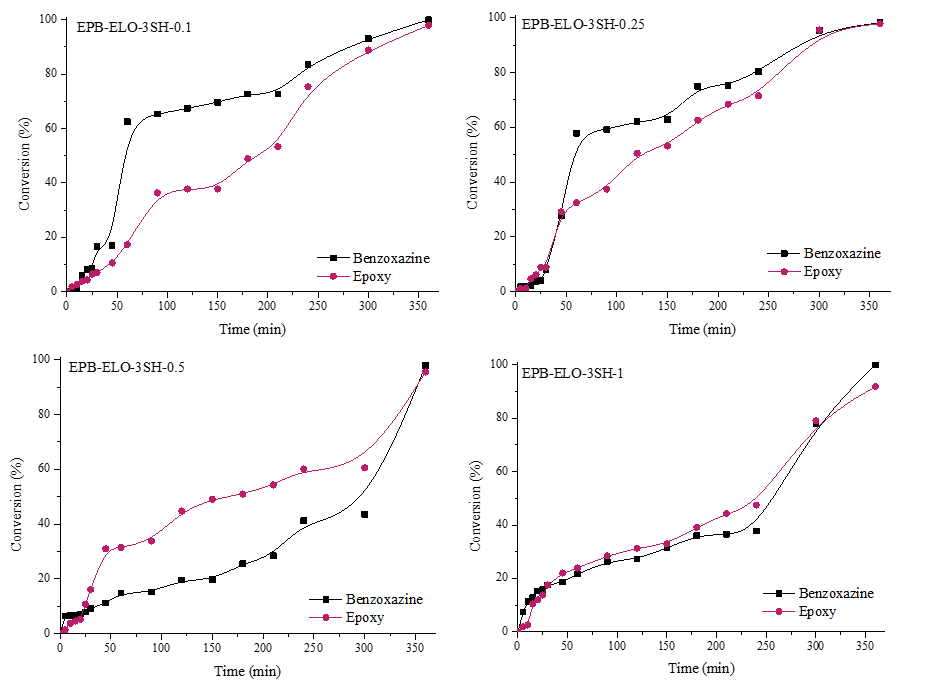


Figure S13. Reaction extent for EPB-ELO-3SH systems.

Figure S14. Comparative curing conversion of benzoxazine moiety as a function of thiol type and concentration

Figure S15. Comparative curing conversion of epoxy moiety as a function of thiol type and concentration


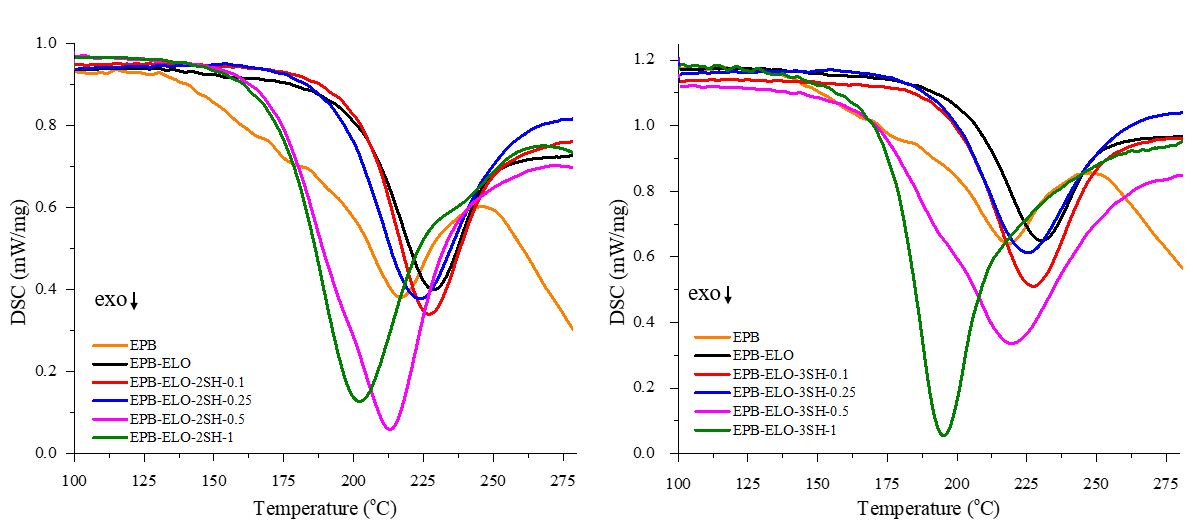


Figure S16. DSC thermogram of hybrid networks

Table S1. DSC parameters for bio-based ternary networks.

| **Sample** | **T_onset_ (°C)** | **T_max_ (°C)** | **ΔH (J/g)** |
| --- | --- | --- | --- |
| ELO | - | - | - |
| EPB | 132.5 | 217.5 | 75.22 |
| EPB-ELO | 184.8 | 229.2 | 139.9 |
| EPB-ELO-2SH-0.1 | 183.6 | 227.7 | 191.8 |
| EPB-ELO-2SH-0.25 | 175.9 | 225.4 | 205.8 |
| EPB-ELO-2SH-0.5 | 156.3 | 213.9 | 391.0 |
| EPB-ELO-2SH-1 | 149.3 | 203.8 | 370.3 |
| EPB-ELO-3SH-0.1 | 180.9 | 226.6 | 230.8 |
| EPB-ELO-3SH-0.25 | 174.7 | 224.6 | 237.2 |
| EPB-ELO-3SH-0.5 | 154.8 | 218.6 | 392.2 |
| EPB-ELO-3SH-1 | 148.3 | 194.8 | 409.8 |


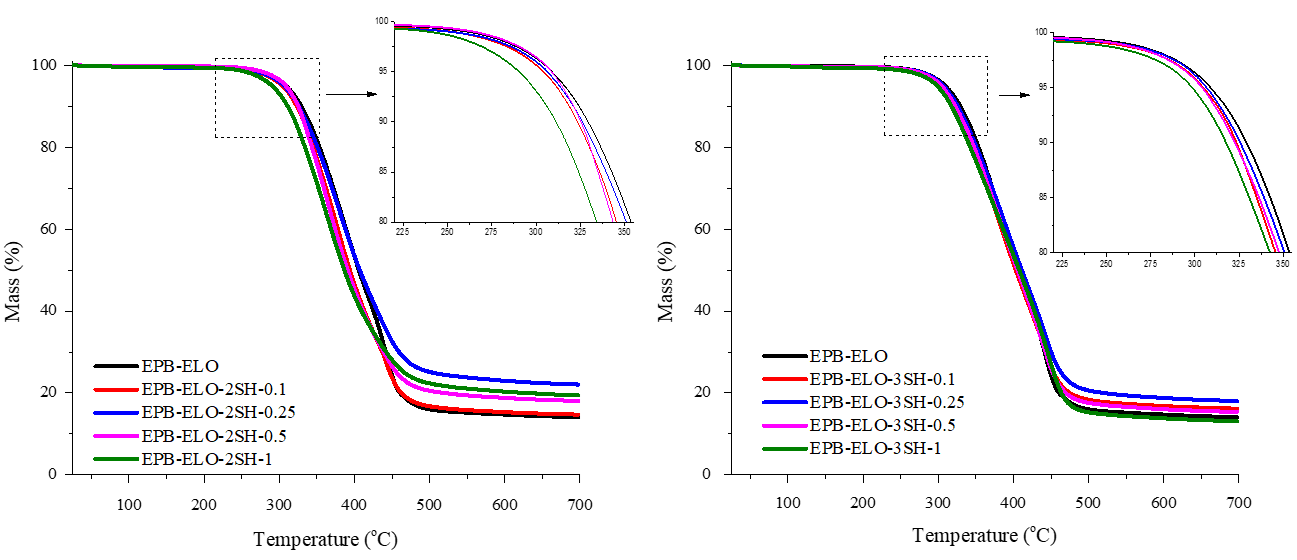


Figure S17. TGA curves for hybrid networks


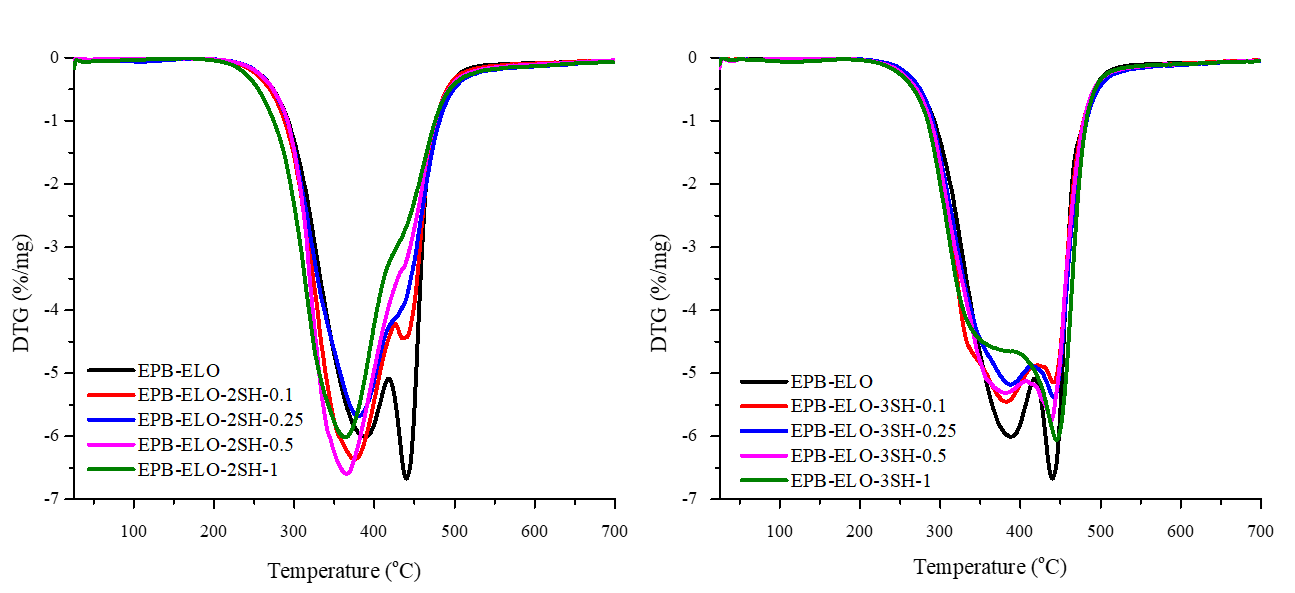


Figure S18. DTG curves for hybrid networks
